# UHPLC–electrospray ionization–mass spectrometric analysis of brain cell-specific glucogenic and neurotransmitter amino acid content

**DOI:** 10.1038/s41598-021-95646-8

**Published:** 2021-08-09

**Authors:** Khaggeswar Bheemanapally, Prabhat R. Napit, Mostafa M. H. Ibrahim, Karen P. Briski

**Affiliations:** School of Basic Pharmaceutical and Toxicological Sciences, College of Pharmacy, University of Louisiana Monroe, 356 Bienville Building, 1800 Bienville Drive, Monroe, LA 71201 USA

**Keywords:** Biochemistry, Biotechnology, Molecular biology, Physiology

## Abstract

Astrocyte glycogen, the primary energy reserve in brain, undergoes continuous remodeling by glucose passage through the glycogen shunt prior to conversion to the oxidizable energy fuel l-lactate. Glucogenic amino acids (GAAs) are a potential non-glucose energy source during neuro-metabolic instability. Current research investigated whether diminished glycogen metabolism affects GAA homeostasis in astrocyte and/or nerve cell compartments. The glycogen phosphorylase (GP) inhibitor 1,4-dideoxy-1,4-imino-d-arabinitol (DAB) was injected into the ventromedial hypothalamic nucleus (VMN), a key metabolic-sensing structure, before vehicle or l-lactate infusion. Pure VMN astrocyte and metabolic-sensory neuron samples were obtained by combinatory immunocytochemistry/laser–catapult-microdissection for UHPLC–electrospray ionization–mass spectrometry (LC–ESI–MS) GAA analysis. DAB inhibition of VMN astrocyte aspartate and glutamine (Gln) levels was prevented or exacerbated, respectively, by lactate. VMN gluco-stimulatory nitric oxide (NO; neuronal nitric oxide synthase-immunoreactive (ir)-positive) and gluco-inhibitory γ-aminobutyric acid (GABA; glutamate decarboxylase_65/67_-ir-positive) neurons exhibited lactate-reversible asparate and glutamate augmentation by DAB, but dissimilar Gln responses to DAB. GP inhibition elevated NO and GABA nerve cell GABA content, but diminished astrocyte GABA; these responses were averted by lactate in neuron, but not astrocyte samples. Outcomes provide proof-of-principle of requisite LC–ESI–MS sensitivity for GAA measurement in specific brain cell populations. Results document divergent effects of decreased VMN glycogen breakdown on astrocyte versus neuron GAAs excepting Gln. Lactate-reversible DAB up-regulation of metabolic-sensory neuron GABA signaling may reflect compensatory nerve cell energy stabilization upon decline in astrocyte-derived metabolic fuel.

## Introduction

Brain neurons expend a significant fraction of total bodily energy in order to sustain critical functions such as maintenance of trans-membrane ion gradients and implementation of cell-to-cell communication. Deficit supply of glucose, the primary energy source to the brain, can impair neurological function and cause injury. Brain cell energy balance is screened unremittingly by specialized metabolic-sensing neurons localized to the ventromedial hypothalamic nucleus (VMN) and a small number of other brain loci^1–5^. Neurochemicals that mediate effects of ventromedial hypothalamic energy imbalance include the amino acid γ-aminobutyric acid (GABA), which suppresses glucagon and epinephrine responses to hypoglycemia^6^, and the gaseous transmitter nitric oxide (NO), which is stimulatory to counter-regulatory hormone release^7^. VMN GABAergic and nitrergic neurons are an evident direct source of information on cellular energy stability as both nerve cell types source express the ultra-sensitive energy gauge 5′-AMP-activated protein kinase (AMPK)^8,9^.

Brain astrocytes are a vital contributor to neuronal metabolic stability as these glia take up glucose from the circulation, amass this energy substrate in the form of the complex polysaccharide glycogen, and convert glucose to the oxidizable metabolic fuel l-lactate^10^. Lactate is transferred between astrocytes and neurons by cell type-specific monocarboxylate transporters to sustain mitochondrial ATP production in the latter cell compartment^11^. Glycogen metabolism is regulated by opposing glycogen synthase and glycogen phosphorylase (GP) enzyme actions, which catalyze glycogen synthesis or disassembly in that order. Astrocyte glycogen undergoes constant remodeling during metabolic homeostasis, and provides needed lactate equivalents during states of increased neurological activity or glucoprivation^12^. A significant fraction of glucose internalized by astrocytes passes through the glycogen shunt, which involves sequential glucose incorporation into and liberation from this polymer, prior to entry into the glycolytic pathway^13,14^. Stereotactic administration of the GP inhibitor 1,4-dideoxy-1,4-imino-d-Arabinitol (DAB) to the VMN stimulates expression of neuronal nitric oxide synthase (nNOS), a marker protein for NO transmission^15^. Those results imply that decrements in astrocyte glycogen-derived substrate fuel stream elicit neurotransmitter signaling of energy insufficiency within the VMN.

There is mounting evidence for brain cell utilization of non-glucose-derived energy during metabolic imbalance. Hypoglycemia-associated reductions in brain glutamine (Gln) and glutamate (Glu) suggest that these glucogenic amino acids may serve as cerebral energy substrates during systemic glucoprivation^16^. The energy-producing glutaminolysis pathway involves conversion of Gln to Glu for entry into the pyruvate recycling pathway, which provides the tricarboxylic acid (TCA) cycle with pyruvate-generated non-glucose substrates^17^. Pyruvate recycling is also fed by the amino acid aspartate (Asp) as aspartate transaminase catalyzes conversion of Asp and α-ketoglutarate to Glu and the TCA cycle component oxaloacetate. Glucose deficits reportedly enhance nerve cell pyruvate recycling pathway activity^18^. It remains unclear whether or how glycogen metabolism may affect astrocyte or nerve cell glucogenic amino acid homeostasis. Laser–catapult microdissection (LCM) enables collection of homogeneous cell samples from tissues and organs composed of multiple cell types, including the brain^19,20^. Here, individual VMN astrocytes and metabolic-sensory, e.g. GABAergic and nitrergic neurons were identified by in situ immunocytochemistry prior to LCM procurement from rats treated by bilateral intra-VMN stereotactic administration of the GP inhibitor DAB, accompanied by vehicle or l-lactate infusion. Pure cell population samples were analyzed by UHPLC–electrospray ionization mass spectrometry (LC–ESI–MS) to investigate the premise that VMN glycogen metabolism affects Gln, Glu, and Asp utilization in astrocyte and nerve cell compartments. The amino acid creatine (CR) has a critical role in regulation of intracellular ATP levels due to its capacity for rapid detachment of a phosphate group for binding to ADP. Methodology described above was applied here to measure levels of this energy-binding amino acid in VMN astrocyte and neurons, and to determine how content may be affected by impaired glycogen turnover and diminished substrate fuel stream. This project work also afforded the unique opportunity for validation of utility of LC–ESI–MS for quantification of the inhibitory metabolic transmitter GABA within individual brain cell populations, a critical advance in neuroanatomical resolution relative to our prior work involving LC–ESI–MS measurement of GABA content of VMN tissue acquired by micropunch dissection^21^. Here, discriminative cell type-specific GABA analysis was performed to gain insight on whether DAB-associated adjustments in glucogenic amino acid profiles correlate with changes in signal volume of this inhibitory metabolic neurotransmitter.

## Materials and methods

### Animals

Adult Female Sprague–Dawley rats (2–3 months of age) housed in shoe box cages (2–3 per cage), under a 14 h light/10 h dark lighting schedule (lights on at 05.00 h). Animals were allowed ad libitum access to standard laboratory rat chow (Harlan Teklad LM-485; Harlan Industries, Madison, WI) and tap water, and were acclimated to daily handling prior to experimentation. All animal protocols were conducted in compliance with NIH Guide for Care and Use of Laboratory Animals, 8th Edition, under approval by the ULM Institutional Animal Care and Use Committee. All studies were performed in accord with ARRIVE guidelines. On study Day 1, rats were anesthetized with ketamine/xylazine (0.1 mL/100 g *bw*; 90 mg ketamine:10 mg xylazine/mL; Covetrus, Portland, ME), and with a 26-gauge double stainless-steel cannula guide (prod no. C235G-1.2/SPC; Plastics One, Inc., Roanoke, VA) aimed at the VMN (coordinates: 2.85 mm posterior to *bregma*; 0.6 mm lateral to midline; 9.0 mm below skull surface) by automated stereotaxic surgery^22^. During surgery, animals were bilaterally ovariectomized (OVX) and implanted with a subcutaneous (*sc*) capsule (10 mm/100 g *bw*; 0.062 in. *i.d*, 0.125 in. *o.d*.) filled with 30 µg 17β-estradiol-3-benzoate/mL safflower oil. This steroid replacement regimen yields approximate plasma estradiol concentrations of 22 pg/mL^23^, which replicate circulating hormone levels characteristic of 4-day estrous cycle metestrus in ovary-intact female rats^24^. After surgery, rats were injected with enrofloxacin (Enroflox 2.27%; 10 mg/kg *bw IM*) and ketoprofen (3 mg/kg *bw sc*), then transferred to individual cages.

### Experimental design

Animals were divided into three groups (n = 4/group). At 0.900 h on day 7, rats were injected using a double internal injection cannula (C235I-1.2/SPC; Plastics One; 1.0 mm projection) with vehicle (V) (sterile 0.9% saline; SAL; group 1) or DAB (150 pM^25^; groups 2 and 3) in a total volume of 0.5 uL, at a rate of 0.25 µL/min over a 2 min period. After treatment, the internal cannula was kept in place for 60 s. Beginning at 09.10 h, animals were continuously infused with V (groups 1 and 2) or l-lactate (100 nM^25^; group 3), in an infusion volume and rate similar to that described above. At 10.00 h, brains were collected at sacrifice by microwave fixation (1.45 s exposure; In Vitro Microwave Fixation System, 5 kW; Stoelting Co., Wood Dale, IL)^9,21^, snap-frozen in liquid nitrogen-cooled isopentane, and stored at − 80 °C.

### VMN neuron and astrocyte laser–catapult microdissection

A consecutive series of 10 μm-thick frozen sections were cut over the rostro-caudal length of the VMN and mounted on PEN membrane-coated slides (Carl Zeiss Microscopy, LLC, White Plains, NY). Tissues were fixed with acetone, washed with phosphate-buffered saline, pH 7.5, containing 0.1% Tween 20, then blocked with 2.5% normal horse serum (prod. no. MP-7800; Vector Laboratories, Burlingame, CA). Sections were incubated for 48 h at 4 °C with rabbit anti-glutamate decarboxylase_65/67_ (GAD) (prod. no. ABN904, 1:1000; Millipore Sigma, Burlington, MA), rabbit anti-neuronal nitric oxide synthase (nNOS) (prod. no. NBP1-39681, 1:1000; Novus Biologicals, LLC, Littleton, CO)^26^ or mouse anti-glial fibrillary acidic protein (GFAP) antibody (1:500; prod. no. 3670S; Cell Signaling Technology, Inc., Danvers, MA)^27^. GAD- or nNOS-immunoreactive neurons were visualized by tissue incubation with ImmPRESS Universal PLUS polymer kit horse secondary antibodies (prod. no. MP-7800; Vector Laboratories, Burlingame, CA), followed by ImmPACT DAB EqV peroxidase substrate kit reagents (prod. no. SK-4103; Vector Lab.). Astrocytes were identified by tissue exposure to Elite ABC biotinylated horse IgG secondary antibody and ABC reagent (Vectastain Elite ABC mouse IgG kit; PK-4002; Vector Laboratories, Inc., Burlingame, CA), and Vector DAB Peroxidase (HRP) substrate kit reagents (SK-4100; Vector Laboratories). A Zeiss P.A.L.M. UV-A microlaser IV (Carl Zeiss Microsci.) was used to individually harvest neurons and astrocytes that exhibited complete labeling of the cytoplasmic compartment. Laser-dissected cells from each animal were collected by cell type into ultrapure water for storage at − 80 °C^21^.

### VMN neuron and astrocyte amino acid LC–ESI–MS analysis

Pre-column sample derivatization, uHPLC column and internal standard selection, and optimized mass spectrometric gas and temperature parameter settings were implemented as previously described^21^. For each treatment group, cell lysate aliquots from individual subjects were combined to create triplicate pools for each amino acid of interest. Briefly, sample pools (100 µL) were derivatized by addition of carbonate buffer, pH 9.0 (100 µL; Spectrum Chemicals Mfg. Corp. New Brunswick, NJ), internal standard (structural analog l-glutamic acid 5-benzyl ester; 50µL), and fluorenylmethyloxycarbonyl chloride (FMOC; 100 µL; Alfa Aesar, Haverhill, MA). 1-Adamantanamine hydrochloride (AD; 50µL; Alfa Aesar) was subsequently added after vortexing and maintenance. Contents were centrifuged, and a clear supernatant was transferred to 350 µL inserts in 2 mL Surestop vials positioned in an auto-sampler tray. An assembled chromatography system [UHPLC Vanquish binary pump (prod. no. VFP10A01/121345), Vanquish auto-sampler (prod. no. VFA10A02/121345), and temperature-controlled Vanquish UHPLC+ column compartment (prod. no. VHC10A02/121345) (ThermoFisherScientific, Waltham, MA)] were coupled to a single quad ISQ EC mass spectrometer (prod. no. ISQECLC/121345) (ThermoFisherSci.). Column and auto-sampler temperatures were 30 °C and 15 °C, respectively. The auto-sampler needle was washed with 10% (v/v) methanol (10 s). ThermoScientific Dionex Chromeleon 7 Chromatography Data System software (prod. no. 7200.0300/121345) was used for mass spectrometric analysis. For negative mode analysis, an Acclaim 120 C18/4.6 mm ID X 100 mm L, 5 µm, 120 Å column (prod. no. 059147; ThermoFisherSci.) was used with a flow rate of 0.25 mL/min and 1 µL injection volume. Mobile phases A and B consisted of 10 mM ammonium acetate and acetonitrile, respectively. During linear gradient mobile phase flow, acetonitrile was increased from 50 to 80% over the initial 4 min, followed by a decline to 50% between 4 and 8 or 15 min. For positive mode analysis, an Accucore Vanquish C18+ column 50 mm L (prod. 27101-052130; ThermoFisherSci.) was used with a flow rate of 0.2 mL/min in linear gradient composition of mobile phase A (60 μL formic acid in 900 mL ultrapure water) and B (acetonitrile) from 50 to 80% over initial 4 min, followed by reduction of acetonitrile by 50% at 8 or 15 min. Optimized mass spectrometric temperature (vaporizer, 250 °C; ion transfer tube, 200 °C) and gas pressure (sheath gas pressure, 25 psig; auxiliary gas pressure, 2 psig; sweep gas pressure, 0.5 psig) were used as described. Total Ion Chromatograms (TIC) of brain cell samples analyzed in negative mode were extracted using different m/z values, e.g. IS, Gln, GABA, and CR at 458.2, 367.1, 324.1, and 352.1, respectively. Positive mode analysis detected IS, Glu, and Asp m/z at 460.2, 369.1, and 355.1 respectively. Amino acid quantification was done using a previously described linear equation^21^. Sample protein content was determined using a ThermoFisherScientific NanoDrop One^c^ microvolume UV–Vis spectrophotometer (840-274200). Brain cell type amino acid concentrations were expressed as ug/mg protein.

### LC–ESI–MS analysis of VMN glycogen tissue content

VMN tissue was obtained by micropunch-dissection from fresh-frozen sections, as described^21^, and transferred to 100 μL 0.02 M Tris buffer, 0.5 M NaCl, pH 7.0. Tissue lysate aliquots from individual subjects were combined within each treatment group to create triplicate sample pools, which were heat-denatured, homogenized by ultra-sonification, and analyzed for protein content by NanoDrop One^c^ UV–Vis spectrophotometer. Supernatant aliquots (20 μL) were hydrolyzed by incubation (2 h) with 10 μL 0.5 mg/mL amyloglucosidase and 10 μL 0.1 M sodium acetate (pH 5.0), followed by sequential heating (100 °C, 5 min) and cooling to room temperature. Hydrolyzed and non-hydrolyzed samples were derivatized with 100 μL 0.5 M 1-phenyl-3-methyl-5-pyrazolone (PMP) reagent supplemented with 0.3 M NaOH. After acidification with 0.75% formic acid (400 μL) and extraction with chloroform, supernatant aliquots (400 μL) was vacuum-concentrated, frozen at − 80 °C, and lyophilized. Lyophilization products were s diluted to 1.0 mL with 10 mM ammonium acetate, bath-sonicated (30 s), and centrifuged. Supernatant aliquots (250 μL) were transferred to 350 μL inserts, which were placed into 2 mL Surestop vials in an autosampler tray. d-(+)-Glucose-PMP was resolved using the Shodex Asahipak NH2P-40 3E column with a mobile phase (75:25 v/v), acetonitrile:10 mM ammonium acetate (0.2 mL/min). d-(+)-Glucose-PMP ion chromatograms were obtained at m/z 510.2 in the aforementioned ThermoFisherScientific Vanquish UHPLC+ System to generate area-under-the curve data^28^. Critical LC–ESI–MS parameters, such as sheath gas pressure (SGP; 25 psig), auxiliary gas pressure (AGP; 4.6 psig), sweep gas pressure (SWGP; 0.5 psig), vaporizer temperature (VT; 150 °C), ion transfer tube temperature (ITT; 150 °C), source voltage (− 2000 V), foreline pressure (1.76 Torr; auto-set by instrument- and variable), source gas (nitrogen; Genius NM32LA 110 V, 10–6520; Peak Scientific, Inchinnan, Scotland), and mass peak area detection algorithm (ICIS/Genesis) were each maintained at optimum^28^.

### Statistics

Mean neuron or astrocyte amino acid measures were analyzed by one-way analysis of variance and Student-Newman-Kuels *post-hoc* test. Differences of *p* < 0.05 were considered significant.

## Results

**Figure 1:**
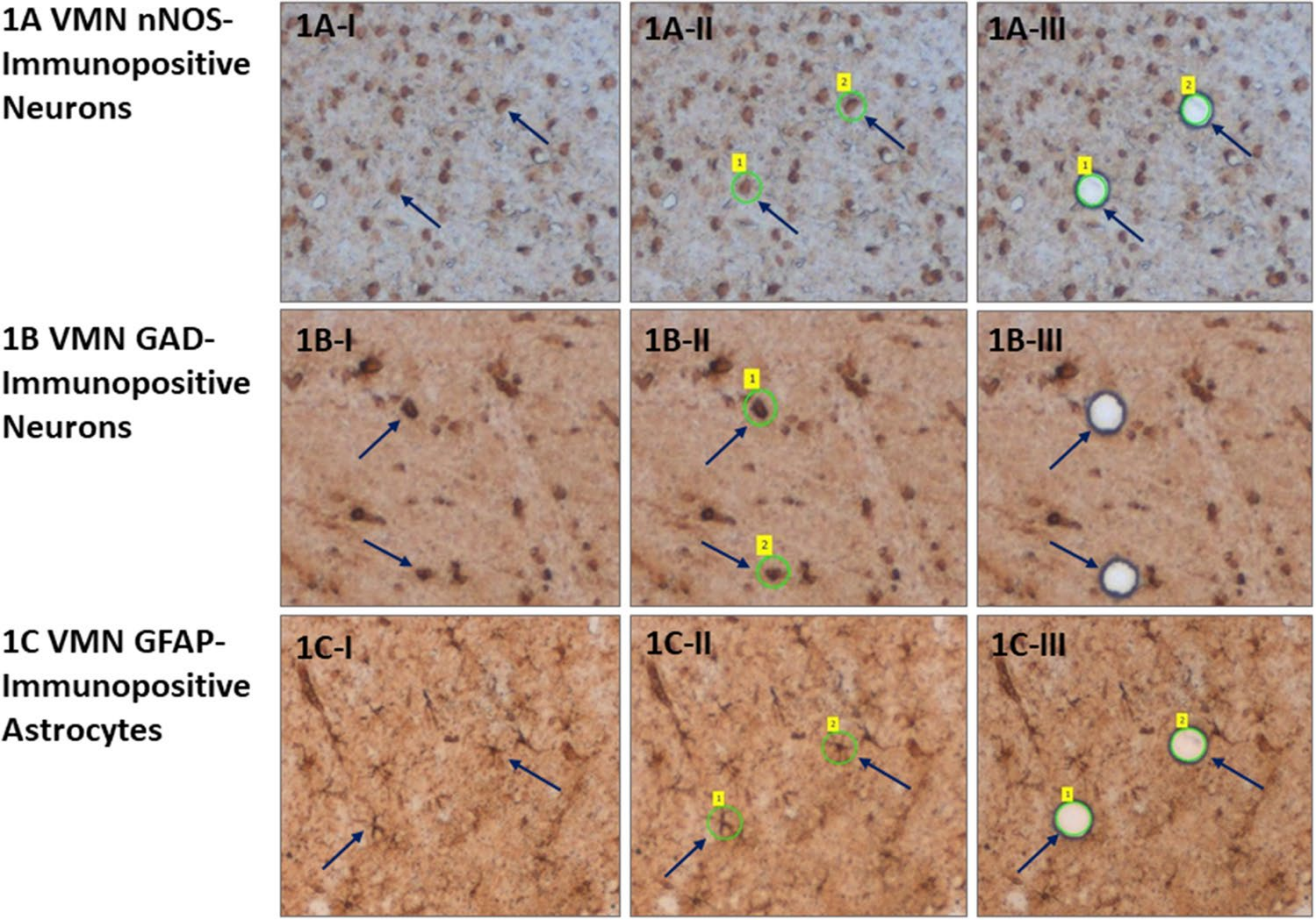
Laser–catapult microdissection of immunolabeled ventromedial hypothalamic nucleus (VMN) astrocytes and metabolic-sensory neurons. Nitric oxide (NO) neurons, and γ-aminobutyric acid (GABA) neurons and. VMN cells were identified in situ for neuronal nitric oxide (nNOS)- (**A**; top row), glutamate decarboxylase_65/67_ (GAD)- (**B**; middle row), or glial fibrillary acidic protein (GFAP)- (**C**; bottom row) immunoreactivity (-ir). Representative nNOS- or GAD_65/67_-ir-positive neurons and GFAP-immunolabeled astrocytes are blue arrows. Areas depicted in Figures (**A-I**), (**B-I**), and (**C-I**), were re-photographed after positioning of a continuous laser track (depicted in green) around individual nNOS-ir (**A-II**; blue arrow), GAD-ir (**B-II**; blue arrow), or GFAP-ir (**C-II**; blue arrow) cells and subsequent ejection by laser pulse (Figures **A-III**, **B-III**, **C-III**). Note that this microdissection technique causes negligible destruction of surrounding tissue and minimal inclusion of adjacent tissue. Scale bars at the bottom right of Figures (**A-III**), (**B-III**), and (**C-III**): 100 µm.

Figure 1 depicts VMN nitrergic neurons (Fig. 1A), GABAergic neurons (Fig. 1B), and astrocytes (Fig. 1C) laser–catapult microdissection. Cells were identified were identified in situ in VMN tissue sections by immunoperoxidase staining for nNOS-, GAD_65/67_−, or GFAP-immunoreactivity (ir), respectively, prior to laser–catapult harvesting [left-hand column; Fig. 1A-I,B-I,C-I]. Blue arrows denote representative labeled nerve or glial cells. Middle and right-hand columns depict sequential actions, e.g. positioning of a continuous laser cut (shown in green in Fig. 1A-II, B-II,C-II) surrounding individual cells, followed by application of a laser pulse, that result in singular cell collection without destruction of surrounding tissue (Fig. 1A-III,B-III,C-III).

**Figure 2:**
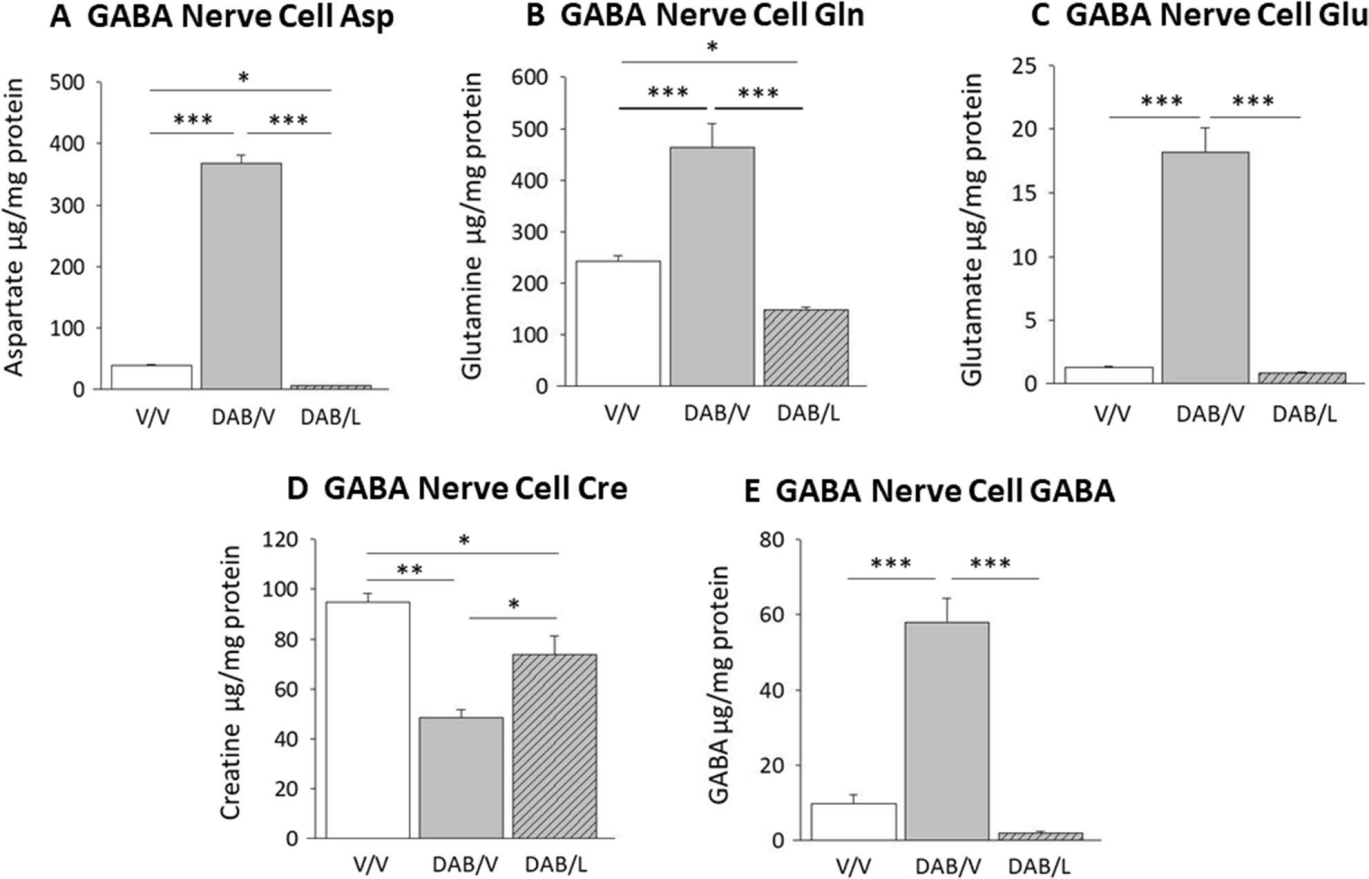
Effects of l-lactate on VMN GABAergic neuron glucogenic and neurotransmitter amino acid concentrations following intra-VMN administration of the glycogen phosphorylase inhibitor 4-dideoxy-1,4-imino-d-arabinitol (DAB). Groups of rats (n = 4/group) were infused (2 min) into the VMN with DAB (150 pM), then infused 10 min later with vehicle (V) or l-lactate (100 nM). For each treatment group, VMN GAD-ir nerve cell lysates from individual subjects were combined to create triplicate samples for LS–ESI–MS analysis of each target amino acids: Data show mean GABA nerve cell aspartate (Asp; **A**), glutamine (Gln; **B**); glutamate (Glu; **C**); creatine (Cre; **D**); and GABA (**E**) amino acid concentrations ± S.E.M. for the following treatment groups: V/V (solid white bars); DAB/V (solid gray bars); DAB/lactate (diagonal-striped gray bars). *p < 0.05, **p < 0.01, **p < 0.001.

Figure 2 depicts effects of intra-VMN administration of DAB followed by V or l-lactate on GABAergic nerve cell amino acid concentrations. DAB caused a significant increase in cellular levels of the glucogenic amino acids Asp (Fig. 2A; F_(2,6)_ = 590.9; *p* < 0.0001), Gln (Fig. 2B; F_(2,6)_ = 56.8; *p* = 0.0044), and Glu (Fig. 2C; F_(2,6)_ = 76.5; *p* < 0.0001) [V/V (solid white bars) versus DAB/V I (solid gray bars)]. Each of these stimulatory responses were prevented by lactate [DAB/L (diagonal-striped gray bars) versus DAB/V (solid gray bars)]. As shown in Fig. 2D, VMN GABA neuron Cre levels were suppressed by DAB, but were normalized in DAB- plus lactate-treated animals [F_(2,6)_ = 14.5; *p* = 0.002]. DAB caused lactate-reversible augmentation of GABA amino acid levels in VMN GABAergic neurons (Fig. 2E; F_(2,6)_ = 58.8; *p* = 0.0001).

**Figure 3:**
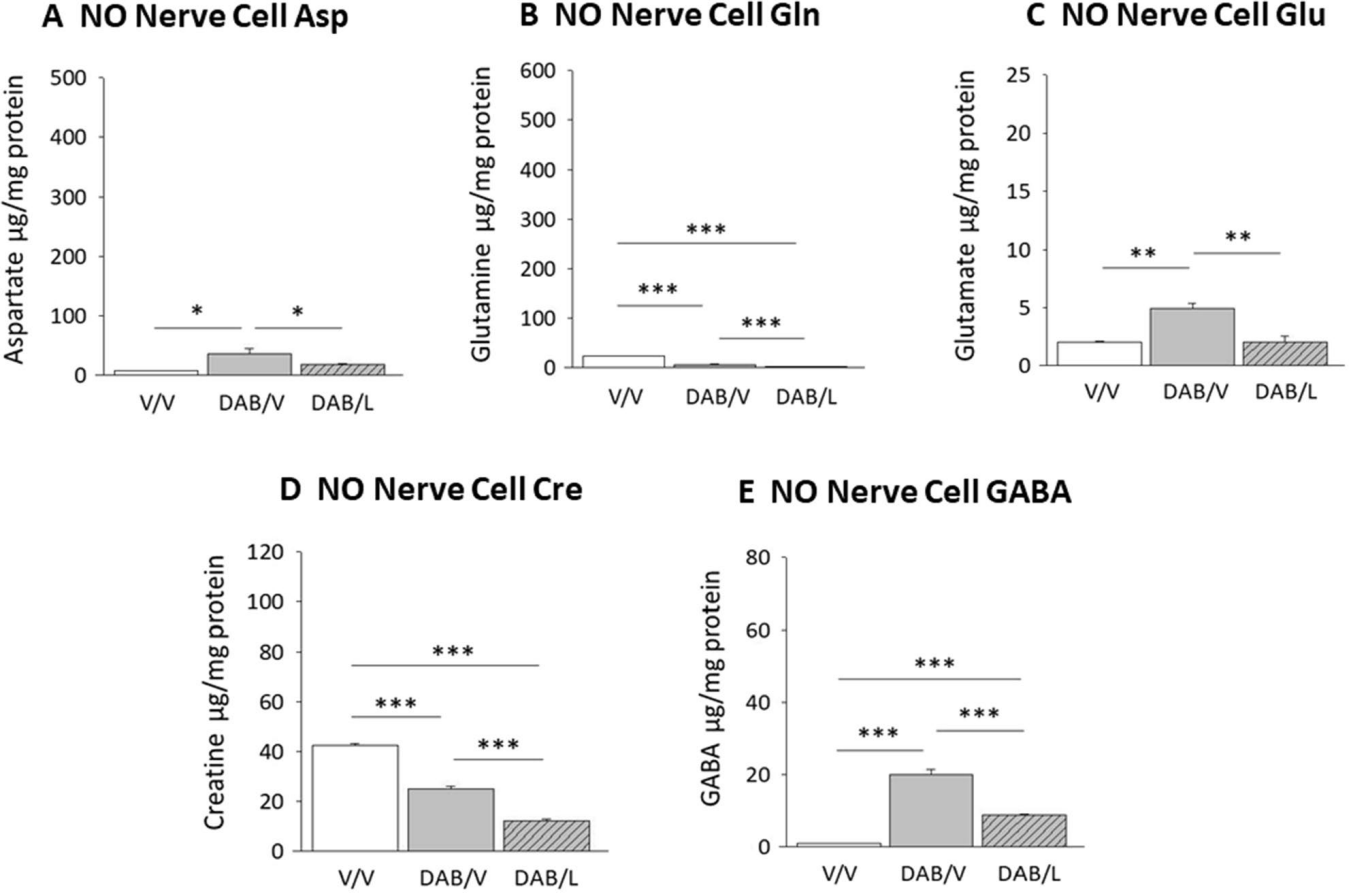
Effects of DAB with or without l-lactate on VMN nitrergic glucogenic amino acid homeostasis. Data show mean NO nerve cell Asp (**A**), Gln (**B**), Glu (**C**), Cre (**D**), and GABA (**E**) concentrations ± S.E.M. for the following treatment groups: V/V (solid white bars); DAB/V (solid gray bars); DAB/lactate (diagonal-striped gray bars). *p < 0.05, **p < 0.01, ***p < 0.001.

VMN nitrergic neurons showed up-regulated Asp (Fig. 3A; F_(2,6)_ = 9.6; *p* = 0.013) and Glu (Fig. 3C; F_(2,6)_ = 18.7; *p* = 0.002) content, but diminished Gln concentrations (Fig. 3B; F_(2,6)_ = 528.7; *p* < 0.0001) in response to DAB. NO nerve cell Cre levels were significantly decreased by DAB, and were further suppressed by DAB plus lactate treatment (Fig. 3D; F_(2,6)_ = 230.2; *p* < 0.0001). DAB increased nitrergic neuron GABA concentrations, a response that was partially reversed by lactate (Fig. 3E; F_(2,6)_ = 127.1; *p* < 0.0001).

**Figure 4:**
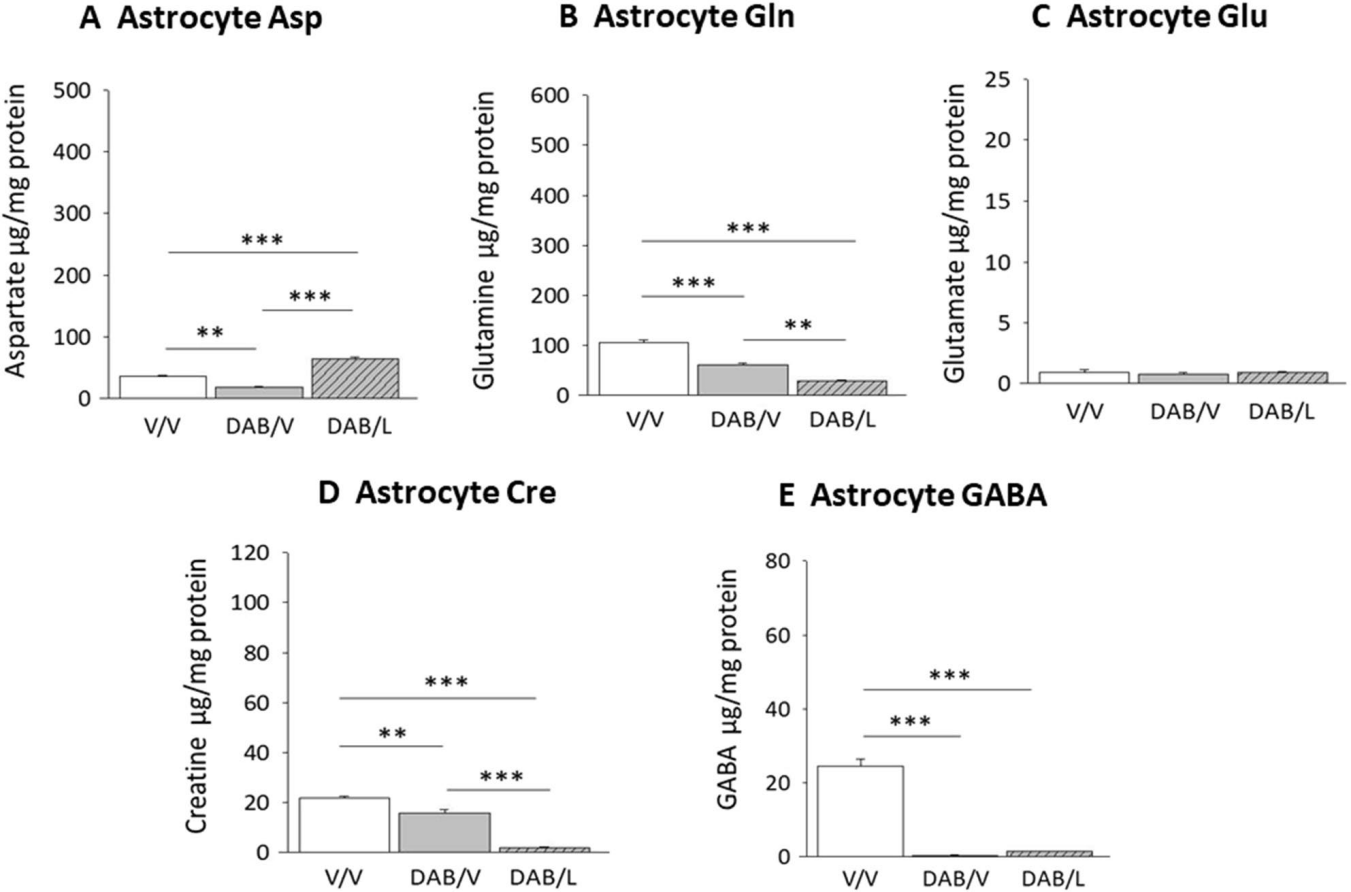
Effects of lactate on VMN astrocyte amino acid concentrations following administration of DAB. Data show mean Asp (**A**), Gln (**B**), Glu (**C**), Cre (**D**), and GABA (**E**) concentrations ± S.E.M. for V/V (solid white bars); DAB/V (solid gray bars); and DAB/Lactate (diagonal-striped gray bars) treatment groups. *p < 0.05, **p < 0.01, ***p < 0.001.

Figure 4 depicts effects of GP inhibition on VMN astrocyte amino acid content. DAB suppressed astrocyte Asp (Fig. 4A; F_(2,6)_ = 71.2; *p* < 0.0001) and Gln (Fig. 4B; F_(2,6)_ = 74.4; *p* < 0.0001) concentrations by lactate-dependent or -independent mechanisms, respectively. Astrocyte Glu levels were refractory to DAB/V or DAB/L treatment (Fig. 4C; F_(2,6)_ = 0.38; *p* = 0.697). DAB suppressed astrocyte Cre content; levels of this amino acid were further diminished by DAB plus lactate [DAB/L versus DAB/V] (Fig. 4D; F_(2,6)_ = 128.3; *p* < 0.0001). Astrocyte GABA content was decreased to a comparable extent by either DAB/V or DAB/L treatment (Fig. 4E; F_(2,6)_ = 149.1; *p* < 0.00010.

**Figure 5:**
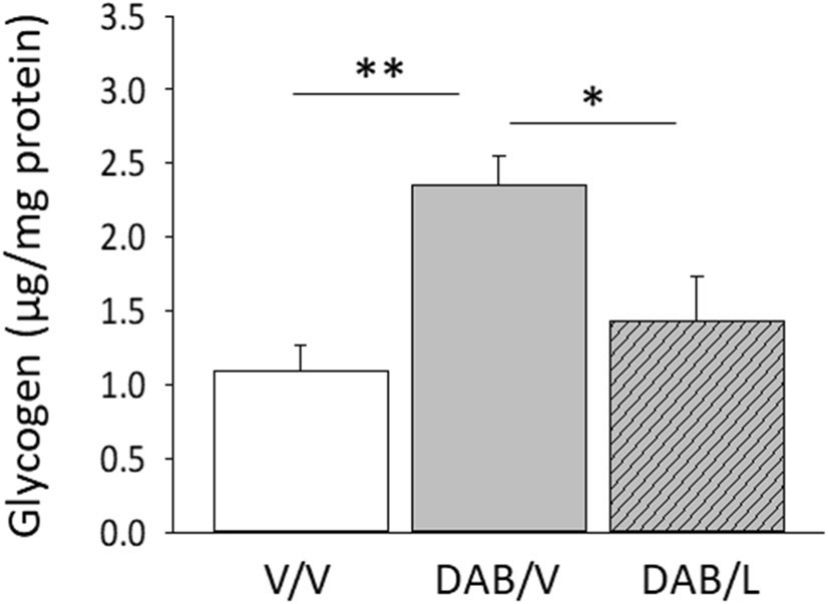
Effects of DAB with or without l-lactate on VMN glycogen tissue concentrations. Data indicate mean VMN tissue glycogen + S.E.M. for the following treatment groups: V/V (solid white bars); DAB/V (solid gray bars); DAB/lactate (diagonal-striped gray bars). *p < 0.05, **p < 0.01.

Data presented in Fig. 5 show that stereotactic-targeted delivery of DAB to the VMN (solid gray bar) resulted in significant augmentation of tissue glycogen content compared to the V/V control group (solid white bar) (F_(2,6)_ = 7.9; *p* = 0.0045). VMN glycogen levels were significantly decreased in animals treated with DAB/L versus DAB/V (diagonal-striped gray bar).

## Discussion

A significant proportion of glucose acquired by astrocytes passes through the glycogen shunt prior to conversion, via glycolytic pathway, to l-lactate. This research investigated the novel premise that disruption of VMN glycogen disassembly affects astrocyte and nerve cell glucogenic amino acid balance by mechanisms involving l-lactate provision. It was also of interest to examine the corollary assumption that DAB-associated changes in Gln, Glu, and/or Asp content may coincide with adjustments in the gluco-inhibitory amino acid transmitter GABA. Outcomes emphasize the efficacy of combinatory high-resolution microdissection and LC–ESI–MS for quantification of baseline and physiological stimulus-associated amino acid profiles in distinct brain cell populations. Results show that DAB up-regulated Asp and Glu in VMN nitrergic and GABAergic neurons, but correspondingly inhibited or did not affect net astrocyte Asp or Glu homeostasis. Attenuation of cell type-specific amino acid responses to GP inhibition by exogenous lactate infers that treatment effects involve diminution of glycogen-derived energy substrate supply. DAB-mediated up- (metabolic-sensory neurons) or down-regulation (astrocytes) of the gluco-inhibitory amino acid transmitter GABA may indicate, in the wake of diminished VMN glycogen breakdown, compensatory nerve cell energy stabilization, involving in part energy production from glucogenic amino acids, whereas astrocytes may adopt a more negative energy state.

Results provide novel evidence that steady-state Asp, Gln, and Glu levels differ not only among VMN astrocytes versus metabolic-sensory neurons, but also, quite unexpectedly, vary between GABAergic and nitrergic nerve cell populations. These findings support the broad assumption that these amino acids may be utilized for a unique number or set of purposes in the three brain cell types examined here, which may contribute to distinctive functionality of each cell type. For each cell population, it is noted that measurable amino acid concentrations at equilibrium do not shed light on their subcellular distribution between a labile nitrogen pool available for rapid use versus incorporation into the polypeptide backbone of cellular structural or enzyme proteins, or the relative mass of Glu that is sequestered in secretory vesicles for stimulus-induced exocytotic release. Further research is warranted to determine pure brain cell sample volume requirements for LC–ESI–MS analysis of amino acid concentrations in distinct cell components separated by subcellular fractionation. Tools of requisite sensitivity and neuroanatomical resolution for assessment of amino acid turnover rate within distinctive intracellular pools of individual brain cell populations in vivo or in vitro are presently unavailable. Observed differences in levels of the energy-binding amino acid Cre support the notion that net capacity for high-energy phosphate binding is likely cell-type specific, which may reflect differing volumes of phosphate that can be released to generate ATP from ADP. Current findings that VMN astrocytes contain GABA align with prior reports that this transmitter is released from these glial cells elsewhere in the brain^29–31^. However, proof obtained here that GABA is measurable in VMN nitrergic neurons, albeit at substantially lower levels relative to GAD-immunoreactive nerve cells, was unforeseen. While it is well established that neurons that co-express multiple transmitters, including GABA, occur in the brain^32–36^, there has been a lack of active contemplation of the possibility of co-release of gluco-regulatory transmitters. Current work advances the novel concept that NO and GABA, which impose pharmacological evidence-based antagonistic effects on glucose counter-regulation, are co-localized in VMN nitrergic neurons. These findings raise a number of important new issues that will require future attention, including whether these disparate signals are controlled by common and/or unique cues that may include systems-level neural circuitry activity and prior exposure to neuro-energetic instability; if and how NO and GABA release may be integrated over time within a specific physiological context, including metabolic homeostasis versus imbalance; and whether these transmitters regulate the same or different post-synaptic targets. Diffusional release of the gaseous transmitter NO is likely to affect closely-approximate neighboring neurons at both synaptic and non-synaptic contact sites, whereas GABA signaling is achieved via synaptic coupling. Thus, these neurochemicals may regulate common as well as unique targets. The notable, approximately tenfold difference in steady state GABA levels measured in GABAergic versus nitrergic neurons may likely reflect a relatively greater negative tone imposed by the former cell population on VMN gluco-regulatory circuitries.

Methodology optimized in our laboratory^28^ for quantification of glycogen in small-volume brain tissue samples was used here to verify effects of targeted delivery of the GP inhibitor DAB on VMN glycogen metabolism. Results document the efficacy of the current DAB dosing paradigm for augmentation of glycogen concentrations in this structure. Data also disclose a reduction in tissue glycogen levels following DAB/L versus DAB/V treatment. Notably, DAB inhibition of glycogen accumulation is insignificant compared to controls in the presence of exogenous lactate. Those results infer that lactate may decrease the ratio of glycogen synthase (GS) versus GP activity relative to effects of DAB alone. A plausible interpretation of this outcome is that lactate abundance alongside amplification of the glycogen reserve may decrease glucose incorporation into glycogen by GS. The notion that GS may be governed by glycogen-derived substrate fuel is supported by recent evidence that norepinephrine-induced suppression of GS profiles is attenuated by monocarboxylate transporter inhibition^37^. Another scenario is that lactate repletion at current dose levels may enhance GP enzyme activity by regulation of phosphorylation state and/or allosteric effectors, to an extent that glycogen breakdown may occur at control-like levels in the presence of DAB. Both aforementioned premises will require experimental verification.

Outcomes here document the direction and magnitude of change in glucogenic amino acid content in specific VMN cell types caused by suppression of glycogen breakdown. The GP inhibitor DAB had dissimilar effects on Asp and Glu levels in neurons versus astrocytes, as these amino acids were both increased in nitrergic and GABAergic neurons, whereas astrocytes exhibited reductions in Asp, but no change in Glu content in response to this treatment. These findings infer that these amino acids may be expended for different purposes or utilized at divergent levels for similar needs, for example, metabolic pathways for production of non-glucose-derived energy. Further research is required to determine how labile pools of these amino acids are partitioned in response to decreased glycogen metabolism. The efficacy of exogenous lactate to reverse these cell type-specific amino acid responses to DAB infers involvement of metabolic fuel stream in astrocyte glycogen metabolism regulation of Asp and Glu homeostasis in these distinctive cell compartments. It is presumed here that glycogen metabolic status is communicated to metabolic-sensory neurons via volume of trafficked lactate. VMN nitrergic and GABAergic neurons express the energy gauge AMPK; it would be useful to learn in future if adjustments in nerve cell amino acid equilibrium triggered by deficient lactate provision involve AMPK activation in those cells. Astrocytes exhibit a rapid capacity to take up lactate from the brain extracellular space^38^. Data here show that exogenous lactate averts DAB-associated decrements in astrocyte Asp content, inferring that Asp equilibrium in these glia may be controlled, in part, by direct actions of lactate. However, evidence here that glycogen mass is reduced by lactate in DAB-treated rats supports the alternative possibility that lactate regulation of Asp levels is secondary to effects on glycogen metabolism.

Disruption of glycogen metabolism elicited divergent changes in VMN GABAergic versus NO neuron Gln content, as this profile was elevated or reduced by GP inhibition in these respective nerve cell populations. Data implicate diminished glycogen-derived lactate supply in DAB-induced augmentation of GABA cell Gln levels, but not Gln decrements in nitrergic neurons. DAB similarly decreased astrocyte Gln content by lactate-independent mechanisms. As an alternative to lactate, NO neuron and astrocyte Gln homeostasis may be responsive to alternative cues on glycogen metabolism, such as net glycogen mass, glycogen synthase activity, and/or branching/debranching enzyme functions that are directly or indirectly affected by DAB. Exacerbating effects of lactate on DAB suppression of NO nerve cell and astrocyte Gln support the notion that these distinctive cell types may be responsive to glycogen-derived signals of energy surfeit that are amplified here by DAB. Further research is warranted to ascertain effects of impaired glycogen metabolism on pyruvate recycling pathway activity in these cell types versus GABA neurons and on intracellular ATP levels. Current data also show that lactate-dependent or -independent mechanisms are similarly involved in DAB effects on GABA neuron versus nitrergic and astrocyte cell Cre content. It remains to be determined if amplifying effects of lactate on DAB-associated Gln and Cre levels in the latter cell compartments involve a causal relationship between these amino acid profiles. A definitive explanation of data that show attenuating effects of lactate on DAB suppression of VMN GABAergic nerve cell Cre content, alongside evidence for lactate exacerbation of drug-associated diminution of this amino acid in nitrergic neurons and astrocytes. It could be speculated that lactate abundance in the presence of augmented glycogen mass may result in decreased Cre levels as a mechanism to reduce, in a cell type-specific manner, capacity for ATP storage; this notion will require experimental verification.

Outcomes provide novel proof that GP inhibition causes lactate-reversible augmentation of GABA content in VMN GABAergic and nitrergic neurons, whereas DAB suppression of this signaling molecule in astrocytes is unaffected by lactate. Lactate may thus attenuate DAB enhancement of nerve cell GABA levels by either direct action on that cell type, or alternatively, by diminution of glycogen mass by possible up-regulation of GP activity. Since earlier work documented a lack of effect of DAB on VMN tissue GAD protein expression^15^, cell type-specific quantification of GABA may provide more definitive insight regarding transmission status than Western blot analysis of biosynthetic enzyme expression profiles. Pharmacological studies involving GABA receptor antagonist delivery to the ventromedial hypothalamus showed that collective GABA activity within that expansive brain region, which contains the VMN and other distinctive neural structures, suppressed counter-regulation^39,40^. Evidence here that VMN gluco-stimulatory nitrergic neurons contain glycogen-sensitive GABA prompts the question of whether or how GABA signaling may modulate control of counter-regulation by this cell type. It is of interest to speculate whether nitrergic neurons receive and integrate distinctive metabolic cues, which are communicated separately to target cells via NO and GABA release, and if GABA may modulate target neuron sensitivity to NO. DAB augmentation of metabolic-sensory nerve cell GABA content infers that these cells may enact compensatory cellular responses to diminished glycogen-derived substrate fuel supply caused by GP repression, including glucogenic amino acid utilization for energy production, toward averting metabolic instability, and that GABA release may signify adaptive maintenance of energy balance.

Present work involved acquisition of nerve cell and astrocyte cell bodies, but not cell processes for analysis of amino acid content. Unfortunately, commercially-available laser–catapult/capture microbeam systems are not outfitted with a beam width of sufficiently small size or an optical microscope configuration that can permit discriminative, high neuroanatomical-resolution dissection of individual extensions derived from a single cell body. In light of recognition that of metabolic functions vary between perikarya and cell processes, including synaptic terminals, current technological inability to evaluate amino acid levels in non-somal cellular domains in situ constrains understanding of how homeostasis may be affected cell-wide by manipulation of glycogen mobilization. It is conceivable that pure primary cultures of brain cell types might allow independent analysis of cell body versus axon terminal amino acid concentrations in vitro. Yet, that alternative approach is hampered by imperfect replication of the in vivo nerve cell microenvironment and, more importantly, involves disconnection of neurons from functional central and peripheral neural systems. Moreover, there is inherent difficulty in generating primary hypothalamic nerve cell cultures, and it is not known at this time if their size and morphology in vitro will permit subcellular analysis. A limitation of the current experimental approach is the lack of insight on dynamic changes in energy substrate metabolism in each cell type of interest. While mass spectrometric imaging is a powerful technique for spatially-defined quantification of a wide variety of molecules and metabolites within a histological tissue framework, it lacks sufficient neuroanatomical resolution required for cellular-level quantification as data were reported for tissue volumes corresponding to brain regions. It is anticipated that future refinements of this valuable approach will permit metabolite imaging in small-size brain areas on a commensurate scale within neuron cell bodies and axon terminals.

While the VMN is a critical component of the brain neuro-metabolic network, it has known structural and functional connectivity with several other hypothalamic loci that participate in energy homeostasis. Outcomes obtained here emphasis the need in future research to evaluate effects of perturbed substrate fuel provision, including that derived from stored glycogen, on the activity state of neurons in those associated structures. It would be informative to learn if neurons that provide afferent innervation to or receive regulatory input from VMN gluco-regulatory nerve cells display equivalent or dissimilar patterns of glucogenic and/or ATP-binding amino acid accumulation during glucostasis versus glucoprivation.

The clear delineation of brain cell types of interest from surrounding elements of neural tissue is an essential requirement for high-precision collection of pure cell samples. Immunocytochemistry was used here for that purpose as it allows for highly accurate cell type identification at the single-cell level. Yet, aqueous processing of brain tissue sections for in situ immunostaining raises the possibility that target amino acids may be artifactually displaced, to an-as-yet-undetermined extent. Amino acids are bound by a superfamily of solute carrier proteins that are present within the plasma membrane and in all cytoplasmic organelles^41^. Amino acid binding to these and other proteins, e.g. enzymes, as well as tRNA serves as the basis for tight regulation of cellular amino acid homeostasis^42^. Such molecular attachments would be expected to minimize amino acid diffusion or loss due to tissue handling during immunolabeling. Future studies could avert potential concern over amino acid dislocation during tissue processing by incorporation of genetic methods for cell type-specific fluorescent protein expression.

In summary, current research demonstrates novel application of LC–ESI–MS for quantification of glucogenic and neurotransmitter amino acids in pure brain cell samples. Results show that VMN metabolic-sensory neurons and astrocytes exhibit dissimilar adjustments in glucogenic amino acid homeostasis, with the exception of Gln, in response to diminished glycogen-derived substrate fuel supply. Divergent changes in GABAergic versus nitrergic nerve cell Gln levels due to GP inhibition infer that the latter neuron population may reactive to non-lactate cues of glycogen metabolism. Data provide novel evidence for co-expression of NO and GABA in VMN nitrergic neurons. DAB-mediated amplification (metabolic-sensory neurons) or suppression (astrocytes) of the gluco-inhibitory amino acid transmitter GABA may indicate, in the wake of diminished VMN glycogen breakdown, compensatory nerve cell energy stabilization, including energy production from glucogenic amino acids, in both nerve cell populations, whereas astrocyte adopt a more negative energy state.

## Abbreviations

Asp: Aspartate
Cre: Creatine
DAB: 1,4-Dideoxy-1,4-imino-d-arabinitol
GABA: γ-Aminobutyric acid
GAD: Glutamate decarboxylase_65/67_
Gln: Glutamine
Glu: Glutamate
GP: Glycogen phosphorylase
LC–ESI–MS: UHPLC–electrospray ioniziation–mass spectrometry
LCM: Laser–catapult microdissection
MBH: Mediobasal hypothalamus
NO: Nitric oxide
nNOS: Neuronal nitric oxide synthase
V: Vehicle
VMN: Ventromedial hypothalamic nucleus
